# Cytotoxic evaluation of *Melia azedarach* in comparison with, *Azadirachta indica* and its phytochemical investigation

**DOI:** 10.1186/2008-2231-21-37

**Published:** 2013-05-16

**Authors:** Samineh Jafari, Soodabeh Saeidnia, Homa Hajimehdipoor, Mohammad Reza Shams Ardekani, Mohammad Ali Faramarzi, Abbas Hadjiakhoondi, Mahnaz Khanavi

**Affiliations:** 1 Department of Pharmacognosy, Faculty of Pharmacy, Tehran University of Medical Sciences, Tehran, Iran; 2 Department of Pharmacognosy, Faculty of Pharmacy, Zanjan University of Medical Sciences, Tehran, Iran; 3 Medicinal Plants Research Center, Faculty of Pharmacy, Tehran University of Medical Sciences, Tehran, Iran; 4 Traditional Medicine and Materia Medica Research Center and Department of Traditional Pharmacy, School of Traditional Medicine, Shahid Beheshti University of Medical Sciences, Tehran, Iran; 5 Department of Traditional Pharmacy, Faculty of Traditional Persian Medicine and Traditional Persian Medicine and Pharmacy Research Center, Tehran University of Medical Sciences, Tehran, Iran; 6 Department of Pharmaceutical Biotechnology, Faculty of Pharmacy, Tehran University of Medical Sciences, Tehran, Iran

**Keywords:** Anti-prolifrative activity, *Azadirachta indica*, Flavonoid, *Melia azedarach*, MTT, Neem, Traditional medicine

## Abstract

**Background:**

*Melia azedarach* L. is an important medicinal plant that is used for variety of ailments in Iranian traditional medicine. *Azadirachta indica* A. Juss is its allied species and possesses similar properties and effects. The present study was undertaken to investigate anticancer activity of these *M. azedarach* in comparison with *A. indica* on cancer cell lines and also to evaluate their safety in humans by testing them on normal cell line. The study also aimed to determine the active components that are responsible for medicinal effects of *M. azedarach* in traditional usages.

**Methods:**

In this study, the cytotoxic activity of crude extracts from *M. azedarach* and *A. indica* leaves, pulps and seeds as well as three main fractions of their leaf extracts were assayed against HT-29, A-549, MCF-7 and HepG-2 and MDBK cell lines. MTT assay was used to evaluate their cytotoxic activities. Methanol leaf fraction of *M. azedarach* as the safest leaf fraction in terms of cytotoxicity was subjected for phytochemical study.

**Results:**

Results of the present study indicated that seed kernel extract of *M. azedarach* had the highest cytotoxic activity and selectivity to cancer cell lines (IC_50_ range of 8.18- 60.10 Î¼g mL^-1^). In contrast to crude seed extract of *A. indica*, crude pulp and crude leaf extracts of this plant showed remarkably stronger anti-prolifrative activity (IC_50_ ranges of 83.45 - 212.16 Î¼g mL^-1^ and 34.11- 95.51 Î¼g mL^-1^ respectively) than those of *M. azedarach* (all IC_50_ values of both plants > 650 Î¼g mL^-1^). The phytochemical analysis led to the isolation of four flavonol 3-*O*-glycosides including rutin, kaempferol-3-*O*-robinobioside, kaempferol-3-*O*-rutinoside and isoquercetin along with a purin nucleoside, Î²-adenosine.

**Conclusions:**

The anti-prolifrative potentials of extracts from different parts of *M. azedarach* and *A. indica* were determined. By comparison, methanol leaf fraction of *M. azedarach* seems to be safer in terms of cytotoxicity. Our study shows that flavonols are abundant in the leaves of *M. azedarach* and these compounds seem to be responsible for many of medicinal effects exploited in the traditional uses.

## Background

*Melia azedarach* L. (Meliaceae), commonly known as Persian lilac or chinaberry, has long been recognized in Iran as a medicinal plant with a variety of medicinal effects and mentioned in ancient medical literatures as *“Azad derakht”*[1,2]. Table 1 presents a review on Iranian ancient literatures on the various traditional uses of different parts of *M. azedarach*. Persian lilac is widely distributed in northern forests of Iran and has been also found to possess outstanding antifeedant, anti-insect and cytotoxic activities. Most of the former studies reported limonoids as responsible compounds for mentioned activities of *M. azedarach*[3,4], *Azadiracta indica* A.Juss (neem), another species from Meliaceae, is a close relative of *M. azedarach*. Neem is originally native to South India and Myanmar. However, it abundantly grows in southern coast of Iran and is popularly known as “*Charish”* there [3,5]. Neem and Persian lilac are very similar in morphology, constituents and properties [6,7] so that they were erroneously mixed with each other many times [1,4]. Neem is similarly known worldwide as commercial natural insecticide, pesticide and agrochemical [5] and is abundant in cytotoxic limonoids [4].

**Table 1:** **Traditional uses of *M. azedarach***

**Plant part**	**Traditional uses**
Flower	Remedy for brain obstructions [2,10-12]
	Temperament normalizer in elderly people and people how suffer from cold dystemperament; headache and other head pains reliever (through inhalation of its sent) [2];
Leaf	Antidote against all type of toxins [2-11];
	Remedy for chronic intestinal obstructions [9-12];
	Purulent sores [2,10];
	Anthelmintic; remedy for kidney stones; treatment of leprosy and vitiligo [2];
	Low back pain reliever; diuretic; emmenagogue [2,12];
	Hair growth inducer [2,9-12];
	Lice killer [2,10-12];
Fruit	Treatment of leprosy and vitiligo; remedy for tinea and head wounds; hair growth inducer [2];
	Phlegmatic fevers and coughs reliever [9].

In Iranian traditional medicine, just whole plants or mixtures of them are used and there is a belief that pure compounds even plant-derived ones have no natural properties like whole plants [8]. Therefore, cytotoxic evaluation of crude extracts can gives us better insight into cytotoxic effects of whole plants. In all studied traditional literatures except one [9], fruits of *M. azedarach* have been mentioned as the plant toxic and fatal part [2,10-12]. It has been stated that they are harmful to the stomach and chest muscles [10,11]. Recent studies also support toxicity of the fruit [13]. Tetranortriterpenes known as meliatoxins have been reported as toxic principles of the fruit [14]. In contrast to the limitation in fruit consumption, leaves of the plant have been prescribed for a variety of indications. Therefore, in order to detailed investigation of the leaves, their main fractions were also studied and the methanol leaf fraction of *M. azedarach* was selected for isolation of active compounds.

In the present study, crude extract of leaves, pulps and seeds of *M. azedarach* and *A. indica* as well as different fractions of their leaves were studied against four cancer cell lines (HT29, A549, MCF7 and HepG2) and one normal cell line (MDBK). It is noteworthy that our study is the first report about cytotoxic activity of *M. azedarach* seeds.

## Methods

### General experimental procedures

UV spectra were recorded on an Optizen 2120UV plus UV/VIS spectrophotometer. NMR was run on a Bruker DRX-500 spectrometer (^1^H, 500 MHz; ^13^C, 125 MHz). Semi-preparative HPLC was carried out with a KNAUERHPLC system (Germany) and Eurospher 100â€“7 RP C18 (250 Ã— 20 mm; Macherey Nagel) column. Silica gel (35â€“70, 70â€“230 and 230â€“400 mesh, Merck) and Sephadex LH20 (Fluka BioChemika, 25â€“100 Î¼m) were used for column chromatography. TLC analysis was performed on Silica gel 60 F_254_ or Silica gel 60 RP-18 F_254S_; Merck plates (10 Ã— 10 cm).

### Plant material

Leaves and fruits of *M. azedarach* and *A. indica* were collected from Gorgan (Golestan Province) and Bandar Abbas (Hormozgan Province) respectively. Then, their voucher specimens were deposited at the Herbarium of Faculty of Pharmacy, Tehran University of Medical Sciences, Tehran, Iran (Voucher No. of *M. azedarach*: 6710 THE; Voucher No. of *A. indica:* 6640 THE).

Collected leaves and fruits were separately dried in shade, at room temperature. The dried fruits were de-husked and decorticated and their seed kernels were separated from their husks and pulps. The leaves, pulps of fruits (together with their husks), and seed kernels of each plant were separately crushed into fine powders.

### Preparation of extracts for cytotoxic assay

Percolation technique by methanol/water (80:20, v/v) at room temperature was used for total extraction of the leaves, pulps and seed kernels of two species. The extraction procedure included three consecutive extractions of 48 hours, using fresh solvent each time. The solvent was evaporated under reduced pressure to dry. Both dried leaf extracts fractionated with n-hexane (in order to remove the fatty materials and chlorophylls), chloroform, ethyl acetate and methanol successively. After evaporating solvents, fractions were completely dried to remove their trace solvents. To prepare different concentrations of each extract (650 or 1000 Î¼g/mL) for cytotoxic assay, DMSO 10% was used.

### Extraction and isolation

The powdered leaves (1.20 kg) of *Melia azedarach* were extracted three times (every 48 hours) with 80% methanol at room temperature. The solvent was evaporated at reduced pressure to yield 332.4 g of a crude extract. This extract was fractionated on silica gel (35â€“70 mesh) using four solvents - hexane, chloroform, ethyl acetate and methanol- successively. The solvent in each extract was completely evaporated under reduced pressure to yield 28.9 g (8.70%), 56.5 g (16.99%), 14.3 g (4.31%), and 220.0 g (66.18%), respectively. Then, to isolate the pure compounds, methanol fraction was subjected to chromatography on a silica gel column (70â€“230 mesh; 10Ã—15 cm) eluted with gradient of AcOEt/MeOH (100% AcOEt to 100% MeOH). The chromatographic process was monitored by TLC. TLC sheets were developed using BAW system (*n*-BuOH/HOAc/H2O, 3:3:1), viewed under UV light and then sprayed with anisaldehyde-sulphuric acid reagent. Similar fractions were pooled together to give five fractions (M1-M5). Fractions M3 (28.7 g), collected with AcOEt/MeOH (5:5), was chromatographed again on a silica gel column (230â€“400 mesh; 5Ã—15 cm) with a gradient of AcOEt/MeOH (9:1 to 1:9) to give three fractions (M3a- M3d). Spraying with anisaldehyde-sulphuric acid reagent revealed a group of yellow spots on the TLC plate of M3b.

This fraction was repeatedly chromatographed over Sephadex LH 20 (eluted with MeOH) and monitored by TLC until separation of yellow spots from non-target compounds (e.g. polysaccharides). Finally, three fractions were obtained: M3b1- M3b3.

Fraction M3b2 (272.7 mg) was fractionated by reversed-phase HPLC with a step gradient of acetonitril/water (9:1, 8:2, 7:3, 1:9) to give the following compounds: **1** (2.3 mg), **2** (101.9 mg), **3** (20.1 mg) and **4** (16.1 mg). M3b3 was purified by Sephadex LH 20 chromatography again to yield compound **5** (14.1 mg);

(1) *9Î²-D-ribofuranosyladenine (Î²-adenosine)*

*Rf* values Ã—100: 52 (system: BAW (*n*-BuOH: HOAc: H2O, 3:3:1)); ninhydrin and dragendrof-positive compound; ^1^H-NMR (DMSO-*d6*): *Î´* 8.34 (1H, *s*, H-8), 8.12 (1H, *s*, H-2), 7.34 (2H, *bs*, NH_2_) 5.85 (1H, *d*, *J* = 6 Hz, H-1'Î±), 5.46 (2H, *bs*, OH-2', OH-5'), 5.25 (2H, *bs*, OH-3'), 4.59 (1H, *bs*, H-2'), 4.12 (1H, *bs*, H-3'), 3.94 (1H, *d*, *J* = 3, H-4'), 3.66 (1H, *bs*, H-5'a), 3.52 (1H, *bs*, H-5'b). ^13^C NMR (DMSO-*d*_6_): *Î´* 156.23 (C-6), 152.49 (C-2), 149.11 (C-4), 140.03 (C-8), 119.41 (C-5), 87.98 (C-1'), 85.99 (C-4'), 73.53 (C-2'), 70.74 (C-3'), 61.75 (C-5').

(2) *Quercetin-3-O-rutinoside (Rutin)*

Amorphous yellow powder, *Rf* values Ã—100: 57 (system: BAW (*n*-BuOH: HOAc: H2O, 3:3:1); ^1^H-NMR (DMSO-*d*_6_): *Î´* 12.52 (1H, *s*, OH-5), 7.55 (2H, *d*, *J* = 8 Hz, H-2' and H-6'), 6.85 (1H, d, *J* = 8 Hz, H-5'), 6.39 (1H, *J* = 2 Hz, H-8), 6.20 (1H, *J* = 2 Hz, H-6), 5.34 (1 H, *d*, *J* = 7 Hz, H-1'' anomeric proton of glucosyl), 4.40 (1H, *bs*, H-1''' anomeric proton of rhamnosyl), 3.06-3.72 (*m*, remaining sugar protons) and 0.99 (3H, d, *J* = 6 Hz, CH3 rhamnosyl). ^13^C NMR (DMSO-*d*_6_): *Î´* 177.36 (C-4); 164.45 (C-7); 161,25 (C-5); 156.65 (C-9); 156.47 (C-2); 148.50 (C-4'), 144.81 (C-3'); 133.30 (C-3); 121.64 (C-6'); 121.18 (C-1'); 116.27 (C-5'); 115.27 (C-2'); 103.88 (C-10); 101.24 (C-1''); 100.80 (C-1'''); 98.82 (C-6); 93.69 (C-8); 76.46 (C-3''); 75.92 (C-5''); 74.11 (C-2''); 71.87 (C-4'''), 70.59 (C-2'''), 70.41 (C-3'''), 70.02 (C-4''); 68.31 (C-5'''), 67.04 (C-6''); and 17.80 (C-6''').

(3) *Kaempferol-3-O-robinobioside*

Amorphous yellow powder, *Rf* values Ã—100: 60 (system: BAW (*n*-BuOH: HOAc: H2O, 3:3:1)); ^1^H-NMR (DMSO-*d*_6_): *Î´* 12.52 (1H, *s*, OH-5), 8.04 (2H, *d*, *J*= 8 Hz, H-2' and H-6'), 6.86 (2H, *d*, *J*= 8 Hz, H-3' and H-5'), 6.34 (1H, *bs*, H-8), 6.13 (1H, *bs*, H-6), 5.28 (1H, d, *J*= 7.7 Hz, H-1'' anomeric proton of galactosyl), 4.40 (1H, *bs*, H-1''' anomeric proton of rhamnosyl), 3.08 - 3.61 (*m*, remaining sugar protons) and 1.07 (3H, *d*, *J*= 6 Hz, CH3 rhamnosyl).^13^C NMR (DMSO-*d*_6_): *Î´* 177.10 (C-4); 164.4 (C-7); 161.10 (C-5); 160.00 (C-4'), 156.64 (C-9); 156.23 (C-2); 133.23 (C-3); 130.90 (C-2', C-6'); 120.87 (C-1'); 115.08 (C-3', C-5'); 103.4 (C-10); 102.31 (C-1''); 100.07 (C-1'''); 98.8 (C-6); 94.06 (C-8); 73.49 (C-5''); 73.03 (C-3''); 71.92 (C-4'''), 71.12 (C-2''), 70.42, 70.62 (C-2''',C-3'''), 68.29 (C-5'''), 68.01 (4''); 65.32 (C-6''); and 17.92 (C-6''').

(4) *Kaempferol-3-O-rutinoside*

Yellow crystals, *Rf* values Ã—100: 65 (system: BAW (*n*-BuOH: HOAc: H2O, 3:3:1));^1^H-NMR (DMSO-*d*_6_): *Î´* 12.54 (1H, *s*, OH-5), 7.98 (2H, *d*, *J* = 9 Hz, H-2' and H-6'), 6.88 (2H, *d*, *J* = 9 Hz, H-3' and H-5'), 6.38 (1H, *bs*, H-8), 6.18 (1H, *bs*, H-6), 5.30 (1H, *d*, *J*= 7.5 Hz, H-1'' anomeric proton of glucosyl), 4.38 (1H, *bs*, H-1''' anomeric proton of rhamnosyl), 3.04 - 3.86 (*m*, remaining sugar protons) and 0.98 (3H, *d*, *J* = 6 Hz, CH3 rhamnosyl). ^13^C NMR (DMSO-*d*_6_): *Î´* 177.29 (C-4); 165.28 (C-7); 161.21 (C-5); 160.00 (C-4'), 156.78 (C-9); 156.66 (C-2); 133.22 (C-3); 130.94 (C-2', C-6'); 120.94 (C-1'); 115.18 (C-3', C-5'); 103.67 (C-10); 101.50 (C-1''); 100.85 (C-1'''); 99.10 (C-6); 93.98 (C-8); 76.41 (C-3''); 75.76 (C-5''); 74.24 (2''), 71.87 (C-4'''), 70.65 (C-3'''), 70.41 (C-2'''), 69.96 (C-4''), 68.33 (C-5'''); 66.97 (6''); and 17.82 (C-6''').

(5) *Quercetin-3-O-D-glucopyranoside (Isoquercetin)*

Amorphous yellow powder, *Rf* values Ã—100: 70 (system: BAW (*n*-BuOH: HOAc: H2O, 3:3:1)); ^1^H-NMR (DMSO-*d*_6_): *Î´* 12.6 (1H, *s*, OH-5), 7.58 (2H, *d*, *J*= 9 Hz, H-2' and H-6'), 6.84 (2H, *d*, *J*= 9 Hz, H-5'), 6.40 (1H, *d*, *J*= 2 Hz, H-8), 6.19 (1H, *d*, *J*= 2 Hz, H-6), 5.43 (1H, *d*, *J*= 7 Hz, H-1'' anomeric proton of glucosyl), 3.09 - 3.70 (*m*, remaining sugar protons). ^13^C NMR (DMSO-*d*_6_): *Î´* 177.56 (C-4); 164.45 (C-7); 161.33 (C-5); 156.49, 156.35 (C-2, C-9); 148.64 (C-4'); 144.97 (C-3'); 133. 45 (C-3); 121.78 (C-6'); 121.25 (C-1'); 116.29 (C-5'); 115.36 (C-2'); 104.05 (C-10); 101.02 (C-1''); 98.89 (C-6); 93.73 (C-8); 77.65 (C-5''); 76.56 (C-3''); 74.19 (C-2''); 70.01 (C-4''); 61.05 (C-6'').

### Determination of total flavonoid content

The total content of flavonoids was determined using the spectrophotometric method described by Tomczyk *et al.*[15]. The results were expressed as mg of quercetin equivalents (QE) per 100 mg of fraction.

### Cell lines

Four tumor cell lines, HT-29 (human colon adenocarcinoma), A-549 (non-small cell line carcinoma), HepG-2 (hepatocellular carcinoma) and MCF-7 (human breast adenocarcinoma) and one normal cell line, MDBK (bovine kidney cells) were obtained from Pasture Institute of Iran, Tehran, Iran.

All cell lines were grown in suitable media supplemented with 10% fetal bovine serum (FBS) and 1% penicillin-streptomycin and maintained at 37Â°C in a 5% CO_2_ incubator.

### MTT assay

MTT (3-(4,5-dimethylthiazol-2-yl)-2,5-diphenyltetrazolium bromide) colorimetric assay is used to assess cell viability in the presence of different extracts [16]. Cells were seeded into 96-well plates and incubated for 24 h at 37Â°C. Then the medium was replaced with fresh medium containing different concentrations of test extracts. After 72 h incubation at 37Â°C, the medium was changed by fresh medium containing MTT and incubated for additional 4 h. Thereafter, MTT was removed and remaining formazan crystals were completely dissolved in DMSO. Afterwards, the absorbance was recorded at 570 nm, using an ELISA reader. The inhibitory rate was calculated by the following formula:

Relative viability (%) = (Absorbance _test_/Absorbance _control_) Ã—100. IC_50_ value was defined as the concentration of the extract to produce a 50% reduction in viability of cells relative to the negative control (wells exposed to the solvent without any extract). All experiments were performed in triplicate. Tamoxifen was used as positive control.

## Results and discussion

Results of MTT assay were presented in Table 2 as IC_50_ values in Î¼g mL^-1^ and selectivity indexes. Based on this results, the highest anticancer activity revealed in crude seed extract of Persian lilac against HT29 (IC_50_: 8.18 Î¼g mL^-1^), ethyl acetate fraction of neem leaves against HT29 (IC_50_: 18.63 Î¼g mL^-1^) and crude leaf extract of neem against MCF7 (IC_50_: 34.11 Î¼g mL^-1^). It is noticeable that the mentioned extracts displayed relatively higher selectivity to mentioned cancer cell lines compared to tamoxifen. Furthermore, among twelve tested samples, seed kernel extract of *M. azedarach* showed the best cytotoxic activity and selectivity. Hong-Bing *et al*. reported three limonoids and two triterpenes from seed of Persian lilac [17]. Moreover, a new euphane triterpenoid reported by Kelecom *et al*. [18]. Cabral *et al*. showed presence of four lignans in the seed of Persian lilac, which have anti-moulting activity [19]. However, no cytotoxic activity has been reported so far from isolated compounds or crude extract of Persian lilac seeds.

**Table 2:** **Anticancer activity of *M. azedarach *and *A. indica *extracts and fractions on different cell lines**

**Sample**		**Cell lines**								
		**MCF7**		**HepG2**		**A549**		**HT29**		**MDBK**
		**IC**_50_*	**SI**	**IC**_**50**_	**SI**	**IC**_**50**_	**SI**	**IC**_**50**_	**SI**	**IC**_**50**_
**C. Seed E.**	**1**	33.41	1.20	34.91	1.15	60.10	0.67	8.18	4.90	40.13
	**2**	270.58	-	>650	-	370.69	-	343.28	-	>650
**C. Pulp E.**	**1**	>650 **	-	>650	-	>650	-	>650	-	>650
	**2**	139	0.83	212.16	0.54	83.45	1.38	83.49	1.38	115.47
**C. Leaf E.**	**1**	>650	-	>650	-	>650	-	218.61	0.89	195.8
	**2**	34.11	2.06	95.51	0.73	55.84	1.25	38.44	1.82	70.27
**Chloroform F.**	**1**	ND	-	ND	-	ND	-	ND	-	ND
	**2**	38.94	1.13	74.94	0.59	54.59	0.81	46.22	0.95	44.11
**Ethyl acetate F.**	**1**	147.9	0.59	210.3	0.42	146.26	0.60	48.91	1.79	87.56
	**2**	56.29	0.93	49.11	1.07	55	0.95	18.63	2.82	52.55
**Methanol F.**	**1**	>1000***	-	>1000	-	>1000	-	>1000	-	493.81
	**2**	>1000	-	>1000	-	>1000	-	>1000	-	193.3
**Tomoxifen**		3.60	1.22	5.8	0.76	10.7	0.41	2.5	1.79	4.4

*SI* selectivity index, *C.* crude, *E*. extract, *F*. fraction, 1: *M. azedarach* L., 2: *A. indica*, *ND* not detected due to its insolubility;

* IC_50_ values were calculated in Î¼g mL^-1^.

** The sample was not toxic in concentrations less than 650 Î¼g mL^-1^ and insoluble in concentrations more than it.

*** The sample was not toxic in concentrations less than 1000 Î¼g mL^-1^.

The results in Table 2 also indicated that crude pulp and crude leaf extracts of *A. indica,* in contrast to its seed extract, showed remarkably stronger anti-prolifrative activity than those of *M. azedarach*. Moreover, in comparison with Persian lilac, most of neem samples exhibited relatively lower IC_50_ on normal cell line, which indicated that neem is more harmful than Persian lilac and its prescription in high doses need more caution.

It is also noticeable that methanol leaf fractions of both species were inactive against studied cancer cell lines at concentrations blow 1000 Î¼g mL^-1^ and their toxicities on normal line especially that of Persian lilac were less than other leaf fractions. Based on these results as well as the yields of fractions, Persian lilac methanol fraction was selected for active compound isolation. Another point is that traditional herbal medicines are generally prepared as infusions, decoctions, bath formulations and so on in which polar compounds are mostly extracted and lead to pharmacological effects. The methanol fraction as most polar fraction of the leaves is expected to contain foresaid active components more than others.

**Figure 1:**
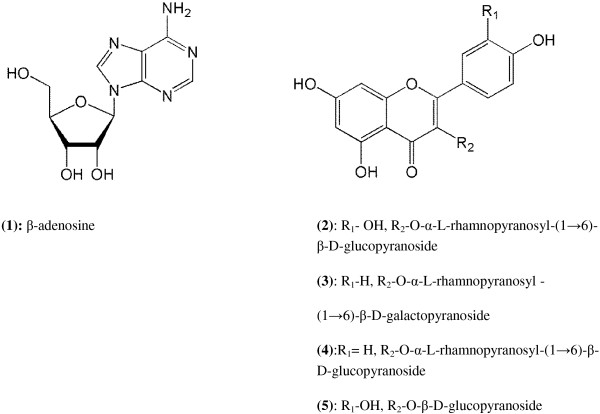
**Molecular structure of the isolated compounds from *M. azedarach.***

The phytochemical study led to the isolation of four flavonoids including rutin, kaempferol-3-O-robinobioside, kaempferol-3-O-rutinoside and isoquercetin along with a purin nucleoside, Î²-adenosine (Figure 1). All the derivatives were identified by comparing their chemical and spectral data with that of published literatures [20,21].

As mentioned, most of previous phytochemical and pharmacological investigations have focused on limonoids and other triterpenoids as active components of *M. azedarach*. Nevertheless, phenolic compounds in particular flavonol glycosides are present in this plant in high levels and it seems that they are significantly involved in the medicinal effects of this plant. Analysis of total flavonoid content in methanol leaf fractions of Persian lilac and neem showed that they both contained high levels of flavonols (72.6 mg QE/100 mg of fraction and 77.8 mg QE/100 mg of fraction respectively). Taking look at the previous reports on medicinal effects of the isolated flavonols shows that they can be responsible for many effects which are listed in Table 1. For instance, flavonols as quercetin, kaempferol and their glycosides can be used as antidote for a broad range of toxic materials as snake venoms [22,23], heavy metals, T2 toxin [24], bacterial toxins (e.g. Microcystin, botulinum neurotoxin) [25,26], mustard [27], bisphenol A [28], arsenic [29]. In addition, rutin and quercetin were reported to ameliorate inflammatory bowel disease [30]. This effect may be associated with the traditional prescription of Persian lilac for chronic intestinal obstructions. Also, rutin and quercetin are able to enhance wound healing [31,32] so that they are effective in treatment of suppurative wounds [33]. Rutin has showed anthelmintic activity against human lymphatic filariasis [34] and *Haemonchus contortus*[35]. Moreover, 3-*O*-glycosides of kaempferol and quercetin exhibited diuretic activity [36] which was found to be mediated by the action at A1 adenosine receptors like caffeine and theophylline [37]. This activity can be a helpful factor in kidney stone treatment too [38]. As a remedy for vitiligo, quercetin has potential to enhance melanogenesis on human epidermis by affecting on maturation of melanosomes [39] and increase the activity and biosynthesis of tyrosinase in melanoma cells and in human melanocytes [40]. Regarding to hair growth, this compound was reported to treat and prevent of alopecia areata [41] and inhibit of 5 Î±-reductase [42].

## Conclusion

Our study determined the anti-prolifrative potentials of extracts from different parts of *M. azedarach* and *A. indica*. Some extracts such as seeds extract of *M. azedarach* are strongly and selectively able to induce cell death of studied cell lines. Methanol leaf fractions were found to be safer in terms of cytotoxicity and abundant in flavonols. Although these plants are famous for their limonoids, it seems flavonols can be in charge for many medicinal effects of leaves of the plants. The present study therefore can provide new context for further researches.

It should be noted that in the present study, cytotoxic assay by means of cell culture as an *In vitro* model cannot assuredly confirm safety or toxicity of the extracts [43]. Although the mentioned plants have been used as traditional medicines for centuries, they are abundant in cytotoxic, insecticide and pesticide compounds. So, performing further *in vivo* animal and human assays is suggested to confirm the safeties of these plants in different aspects.

## Competing interests

The authors declare that they have no conflict of interests.

## Authors’ contributions

SJ performed isolation of plant compounds, reviewed traditional literatures and drafted the manuscript. SS participated in design of phytochemical study and carried out the interpretation of the NMR data and identification of the compounds. HH performed cytotoxic assays and analysis of their data. MRSA was responsible for the conception and design of the research. MAF advised cytotoxic assays and its analysis. AH advised methods for isolation and identification of compounds. MK conceived and designed the study and redacted the manuscript. All authors read and approved the final manuscript.
